# A Practical Manual of Gynæcology

**Published:** 1888-03

**Authors:** 


A Practical Manual of Gynæcology. By G. R. Southwick, M. D. Pp. 408; price, $3.75. Otis Clapp & Son, Boston and Providence. 1888.

The author says in his preface that he has written this manual "as a safe and practical guide for the general practitioner and student rather than for the specialist." He also asserts his belief that uterine, like other diseases, can be cured by internal medication. Why not? we should like to ask. Dr.

Southwick is evidently up with the latest works on his specialty, and has done his work thoroughly, but we should like to see more reliance placed on internal medication and less upon surgery. The indications for the remedies suggested could be much more clearly stated.

